# Pilinahā: An Indigenous Framework for Health

**DOI:** 10.1093/cdn/nzz001

**Published:** 2019-02-22

**Authors:** Sharon Kaʻiulani Odom, Puni Jackson, David Derauf, Megan Kiyomi Inada, Andrew H Aoki

**Affiliations:** 1 Kōkua Kalihi Valley Comprehensive Family Services, Honolulu, HI; 2 Islander Institute, Kailua, HI

**Keywords:** indigenous health, community health, Native Hawaiian, health equity, framework

## Abstract

This article speaks to the abundance and wisdom of indigenous community members in Kalihi, an urban neighborhood in Honolulu, Hawaii. Its findings result from community members sharing their stories of health, health care, and healing. These stories evolved into a distinct framework for health—*Pilinahā* or the Four Connections Framework. Pilinahā addresses 4 vital connections that people typically seek to feel whole and healthy in their lives: connections to place, community, past and future, and one's better self. This article describes the origins, intent, key concepts, and implementation of this framework. By doing so, the authors hope to add to the growing body of work on community and indigenous well-being, further the dialogue with other indigenous communities, and collectively foster a more meaningful and effective health system for all.

## The Larger Context

The larger context of this article is two discrepancies. First, indigenous people come from a lineage of interconnected well-being that is abundant and sustainable (1, 2). Yet health assessments consistently report that the majority of indigenous communities in the United States are sick, with high rates of chronic illnesses (3, 4) that were not historically prevalent (2). Second, despite the tremendous growth in health-related financial spending and technology in the United States, Americans overall do not feel healthier (5, 6). The two discrepancies are related.

Among industrialized nations, the United States continues to spend the most on health care and yet has the worst health and health care outcomes, including for infant mortality, mortality amendable to medical care, and access and equity of care (6). In an effort to address these shortcomings, the health care industry has been exploring the “social determinants of health” (SDoH). The World Health Organization defines SDoH as “the conditions in which people are born, grow, live, work, and play. These circumstances are shaped by the distribution of money, power, and resources . . . [and] are mostly responsible for health inequities” (7).

As indigenous people, we applaud this effort. Guided by ancestral wisdom, we have understood the significance of SDoH factors for generations, not as a passing trend but as a deep cultural, historical, and spiritual understanding of health as wholeness. However, even with this encouraging move toward SDoH, the US health system continues to frame health care as mostly an individual matter between the patient and the doctor and, in its atomizing market approach, discourages indigenous people from valuing ancestral pathways that have successfully healed and sustained our communities for generations. When seeking care at hospitals and private practices, indigenous people tend not to see their gifts and values reflected back (1, 8); instead, we are often blamed for our illnesses, labeled as “noncompliant,” and reproached for neglecting our health (8, 9). This accusatory, deficits-based reasoning is often used to rationalize health inequities in our communities (1, 8, 9, 10). The discrepancy between what we know as indigenous people and what we experience as participants in the health care system inspires us to remember our indigenous practices and reconnect our *lāhui* (nation; in this article, all non-English italicized words are Hawaiian, followed by translations in parentheses) to its traditional sources of healing and health. It motivates us to widen our gaze from biomedical measures of health and illness to our whole story and to listen—first, foremost, and deeply—to the stories of success, struggle, joy, and pain in our community.

Findings in this article are a result of Hawaiʻi community members sharing their accounts of health, health care, and healing with two community-based organizations: Kōkua Kalihi Valley Comprehensive Family Services (KKV) and the Islander Institute. The storytelling surfaced common understandings, issues, and values that in time constellated as a distinct framework, which we have come to call *Pilinahā* or the Four Connections Framework. *Pilina* in Hawaiian means connection or relationship. The Hawaiian language commonly has layered meanings, and here *hā* references *ʻehā*, meaning 4, as well as *hā*, meaning breath of life. Pilinahā represents the 4 connections essential for health and healing. In this article, we share the origins of this framework—what it is, how and why it was created, and what it represents to us. Our hope is to add Pilinahā to the growing body of work on community and indigenous well-being and to encourage others to articulate their own community paradigms of health, wealth, wholeness, and happiness.

Food, nutrition, land, and agriculture were critical components of this process and served as a catalyst, programmatically (as an initiative spearheaded by food- and land-based programs at KKV), socially (as an effective means of building relationships among participants through the preparing and sharing of meals), and culturally (as a pretext for deep ancestral storytelling around food and agriculture). In keeping with our traditional values, we cannot separate nutrition from this overall framework of a community's health; hence, this article by necessity includes this larger scope. We also hope to illustrate how a food and nutrition program, when effectively linked to culture and community, can begin effecting change at an organizational and even systems level, beyond its conventional programmatic domain.

Overall, while listening to our community, we witnessed how the underlying question became not How do we make individuals conform to the system? but, rather, How can we reshape the system to hold a more diverse representation of experiences, whole stories, and healing pathways? One participant shared how “when we place ourselves in a shared story, we become more than just something that needs to be treated.” We hope to uplift these community and indigenous voices to encourage a more meaningful and effective health system for all.

## The Team

KKV is a community-organized and operated Federally Qualified Community Health Center and receives funding through the Health Resources and Services Administration as part of the nation's primary care safety net. We serve the residents of Kalihi, a federally designated Medically Underserved Area in urban Honolulu, Hawaiʻi, and home to more than 50,000 individuals of mostly Asian and Pacific Island descent. This diverse and culturally rich community continues to be the first home for many immigrants and migrants when they move to the state. KKV's more than 200 staff interpret in more than 22 languages and dialects and provide an array of clinical, social, and community-based programs. KKV administers 2 intertwined nutrition-related programs:

- Returning to Our Roots (Roots): Through community engagement in “growing, preparing and sharing” organic and culturally relevant foods, Roots works to increase food sovereignty, strengthen cultural identities, and nurture *waiwai* (abundance and wealth in all its forms). Roots staff and volunteers operate a café and gardens, annually producing more than 6000 pounds (∼2722 kg) of fresh produce that is distributed to the community. In addition, Roots helps reconnect more than 200 Kalihi residents with their land, culture, food, and community through more intensive programming, such as *Ehuola*, an experiential and intergenerational learning initiative that helps *‘ohana* (family) clarify and progress toward their health goals. Currently, more than 20 families participate, cooking and sharing knowledge and stories of their traditional foods.
- Ho‘oulu ‘Āina Nature and Cultural Preserve: In 2004, KKV secured a 20-year lease to steward and sustainably develop 100 acres of state conservation land deep in Kalihi Valley. Dedicated to cultural education and community transformation, this land was named Ho‘oulu ‘Āina, meaning “to grow the land” and “to grow because of the land.” Ho'oulu ‘Āina addresses the health needs of the Kalihi Valley community by strengthening the connection between people and land. The community converges to create a thriving upland resource of forest, food, knowledge, spirituality, and healthy activity. Each year, more than 5000 residents and visitors join in native reforestation, organic gardening, “green job” training, environmental education, and the preservation of cultural and healing practices.

The conception of the formative work supporting Pilinahā was primarily birthed by the director of the Roots program and supported by KKV's executive director and director of Hoʻoulu ʻĀina.

Islander Institute, which was contracted to help facilitate discussions, is a civic enterprise founded to address the most important systemic issues in Hawaiʻi, including education, health, social justice, and land use. Through leadership development, community building, and trusting in the wisdom of island people, Islander Institute works to reinstitute island values such as caring for land, building strong communities, honoring traditions, and living a life of responsibility and *aloha* (reciprocal love). The two facilitators from Islander Institute who co-led each of the discussions have decades of experience in place-based initiatives, planning, land use, public health, policy, and community organizing. Equally important, they have a long-term working relationship with KKV and have successfully collaborated on other projects in the past.

## The Method: Listening

KKV staff began this endeavor not as a formal research study but, rather, as an internal exploration with the intent of identifying whole measures of health appropriate for our community. Thus, this article is a perspectives piece on our findings rather than a report of research results and was not reviewed by any institution ethics review board. However, all participants were informed of our intentions and were inspired to voluntarily contribute their *manaʻo* (thoughts, ideas, knowledge, and opinions) to this effort. They agreed that it was important for us—as health providers, patients, and stakeholders of a community—to collectively talk about our understandings of health and healing, in part because everyone agreed that, as a whole, the US health care system did not represent or accommodate indigenous perspectives of health. **Table 1** captures the differences between mainstream US and indigenous health perspectives, as understood by the participants. Its intention is not to criticize or pit one system against the other but, rather, explain the impetus of this work and highlight the need for a broader framework of health—along with related supportive indicators of success, practices, and services—that can accommodate the full spectrum of strengths, challenges, desires, and needs of diverse communities.

**TABLE 1:** Major contrasting foundational views between existing and indigenous frameworks surrounding health and healing

Existing Framework	Indigenous Framework
**Disintegration** – You are an assembly of parts and conditions	**Wholeness** – A community of people and places
**An individual’s concern** – Health is between you and your doctor	**A family/community concern** – We are part of a community of care
**Exclusive** – Health is based on your status, characteristics and category	**Inclusive** – Health is something that unites us and our ideas
**Deficit and scarcity** – The focus is on the symptoms. The resources to get healthy are insufficient and must be rationed	**Abundance** – Health begins wherever we are at with whatever resources we have, and grows from there
**Weakness** – You are a victim of forces outside of you and a passive recipient of the situation you find yourself in	**Resilience** – Health is positive action that emerges from our struggles and challenges
**Health is a test** – You are either healthy or not	**Health is a life’s journey** moving in a positive direction, even if we never “get there.”
**Defined externally** – An expert tells you how and why you are healthy or not, and what you need to do about it	**Defined internally** – With support we assess how we are doing and what we need in order to feel whole
**Incomprehensible** – It is your job to navigate the jargon, science, payment systems, and rules in order to obtain health	**Relatable** – Our understanding of health comes from common experience

To explore what a broader framework might look like, organizers focused on bringing people together to elicit community perspectives on the meaning of health. To successfully do this, organizers gave great thought to the sequencing of activities, the invitation list, the types of foods to prepare, and the space needed to hold deep and meaningful conversation on indigenous health. What emerged was a powerful framework that speaks to the traditional practices of our community.

### Sample/participants

KKV and Islander Institute held a series of 4 formal gatherings in 2014 (November 7 and 8 and December 10 and 17). The original framework is based on stories collected during these gatherings. Thirty-eight unduplicated adults—25 women and 13 men all currently living in Hawaiʻi—attended. Organizers used purposive sampling to hear the depth and breadth of perspectives of personal and collective health. Purposive, rather than random, sampling is common in qualitative research when rich detailed information is desired. All participants were invited by organizers based on their trusted relationships in the community, decades of experience in their various vocations, and their deep connections to community health and indigenous rights. To help ensure maximum variation in perspectives, organizers used a sampling frame based on individuals’ *1*) vocation, including local farmers, producers, educators, academic professors, and leaders of cultural, health, and youth organizations; *2*) relationship to KKV, including staff, patients, and stakeholders; *3*) relationship to the Kalihi community, including residents, service providers, and advocates; and *4*) connection to the Native Hawaiian community, including individuals of Native Hawaiian descent and/or those representing organizations working with Native Hawaiian communities.

We did not confine our information gathering to these 4 meetings, in keeping with a more indigenous approach to research. The indigenous search for knowledge traditionally happens over multiple generations rather than during preset projects or funding periods. We continued to pilot test and share the framework during the following 4 years at more than 16 meetings, workshops, and conferences with KKV staff, patients, participants, and stakeholders. Gatherings included group discussions with Native Hawaiian elders at a community health center on a neighboring island and with multiple generations from multiple families at a local community center. These follow-up sessions provided additional stories and reactions that refined and solidified the framework and confirmed that the views shared in the initial 4 gatherings accurately reflected those of our community, patients, and program participants. These follow-up activities are discussed in more detail later.

### Setting and conditions of data collection

Before engaging in the work of discussing health, organizers encouraged camaraderie among participants by holding caring space at Hoʻoulu ʻĀina. Each interaction began with an *aloha* or welcoming circle, in which people held hands and shared their names, the place(s) they called home, and an ancestor they wanted to bring to the circle. Participants bonded through *mālama ‘āina* (working the land) and sharing several *‘ai pono* meals (*‘ai* meaning to eat and nourish, and pono meaning righteousness and balance). Participants prepared meals with ingredients that their ancestors enjoyed from land the participants worked together. Our intention was to foster good health through feeding bodies and spirits. Only after the group established trust did we provide a simple discussion prompt: Share a story about the last time you felt healthy. Discussions were held in English, in some cases with participants responding in English and Hawaiian. When responding in Hawaiian, participants would then translate into English for English-only speakers to understand. No audio or visual recordings were made. KKV staff transcribed all group discussions. No quantitative information was collected. Overall, this methodology honored the protocols of our community. The richness of participants’ sharing leads us to believe this type of engagement allowed people to share deeply without feeling studied.

### Analysis

After the 4 gatherings were completed, Islander Institute conducted the analysis. Because this was not an academic research project, the institute employed no formal qualitative data analysis styles or coding protocols. Instead, our analysts, who had previously fostered trusting relationship in the community through their years of work, both facilitated all discussions and then studied deeply the transcriptions for similarities among participant stories. This process most resembles grounded theory principles, as similarities (themes) were informed directly from the stories themselves. Once analysts identified the 4 connections as links among the different stories, they shared the connections with the core organizers to ensure accuracy and appropriateness. Both Islander Institute and KKV discussed and agreed on all themes during an ongoing series of face-to-face meetings and emails during the following 6 months. Organizers continued to test and discuss the strengths, weaknesses, questions, and growth of the framework—both collaboratively and separately—during a 3½- year period, up until the writing of this article, over email and during many additional formal and informal gatherings.

## The Framework: Pilinahā

When participants shared their stories of good health, they referred consistently to feeling connected. When sharing stories of poor health, they recalled momentary or chronic loss of connection. Participants spoke about health practices that honor concepts of wholeness and inclusiveness. Health for them is a journey—a series of positive actions that emerged from their struggles and challenges and that the abundance and resilience of community enabled. In their practice of health, they may move forward or backward, but always they desire to move toward greater degrees of connection within themselves and among their community of care, which includes doctors, families, neighbors, ancestors, and places. When we acknowledge the importance of these connections, communities, rather than individuals or symptoms, are the smallest unit of health (11). Such practice accommodates many perspectives on what is needed to feel whole and healthy. It provides more opportunities for people to view themselves as agents rather than recipients of health. It also makes health more accessible rather than enclosing it behind seemingly impassable health industry walls of scarcity and deficiency. Pilinahā helps hold a broader definition of health by addressing 4 vital connections that people seek to feel whole and healthy in their lives:

- **Connection to place**—to have a kinship with ‘āina
- **Connection to community**—to love and be loved; to understand and be understood
- **Connection to past and future**—to have *kuleana* (a purpose in the world)
- **Connection to your better self**—to find and know yourself

The ideas presented in the following discussion are from participants, including direct quotations.

*Connection to place* is to know, touch, steward, and love the lands, seas, and winds of our ancestors. *‘O ka hā o ka ‘āina, ke ola o ka po‘e* (the breath of the land is the life of the people) is a core belief of our land-based programs. Touching, sitting quietly in, or having a conversation in a place that reminds people of home can be spiritually healing in and of itself. One woman explained, “When my father returns home after a long trip the first thing he does to reconnect is not visit with his children, but jump in the ocean.” Other ways of deepening connection to land include *mālama* ‘*āina* (caring for and healing the land) and eating traditional foods grown from land that we *mālama*. When we eat with intention and are “more aware of what we put into our mouth . . . we have a stronger understanding of our responsibility to create a healthy food culture in our family.” Questions that providers, front-line staff, evaluators, and researchers might use to help *assess connection to place* include the following:

- What is the story of the place you call home?
- What places are special to you?
- Can you access the places you need to be in?

**Supplemental Appendix A** documents more examples of questions and practices for each domain.

*Connection to community* refers to the love, intimacy, trust, security, familiarity, and happiness shared with others. In many indigenous cultures, community encompasses relationships with family, friends, neighbors, and land. However, the notion of community is richer than just a group of people because it also speaks to the abundance held in the communal. Originally, this section was coded as “connection to others”—terminology that was approved by our team and subsequently used in many presentations and workshops with KKV staff, community members, and other stakeholders. During this time, no one raised concerns or opposed this coding. However, when our artist was redrawing and painting the framework image for this article, person after person responded negatively to this term as “othering” and “exclusive.” Our team reconvened to address this new reaction of community members who did not want to describe themselves as separate from one another. We were inspired by the following question: How can we describe that we are parts of a whole without breaking us apart into “others”? To help us understand what to do, we went back to community members and presented them with 2 frameworks that were the same shape and color, but 1 included the term “community” and 1 included “others.” Several people preferred the framework with “others,” explaining, “I like the word others because it makes you think about the things you are connecting to.” However, the majority of respondents explained that they resonated more with the word community, that community “included the idea of others, but was bigger,” and that “everyone knows what ‘community’ means but they won't know what ‘others’ means.”

We see the beauty and pitfalls of both terms. Needing to make a decision, we choose to rename this section “community.” All of the foundational stories still speak to this new word. For example, a father shared, “If I cannot talk to my kids, why am I trying to be healthy?” Our relationships to community allow us “to create memories, to remember, to become hungry, and feed each other's hunger.” Practices to deepen this connection include generosity and taking time to talk deeply with others. Questions to help access connection to community include the following:

- Do you feel good about the people you work with?
- Who are the people you eat with?
- Have you told someone you love how much they mean to you?

In Hawaiian, the word for future is *wā ma hope*, meaning time past. It refers to the knowledge that our future depends on our history. In Pilinahā, *connection to past and future* represents the idea that we are all part of a cultural strand. “Our genealogies makes us whole, it's an unbroken chain of stories and practices.” The practices and knowledge of our *kūpuna* (ancestors) help guide our present actions, which in turn affect future generations. “We teach [this concept] in our indigenous birthing class. That the food the family eats, the words they use, their practices, bathe the sperm and egg inside of them, which hold the sperm and eggs of their great-grandchildren, and all their future generations.” By carrying on traditional practices and having cultural understanding and appreciation, we deepen our connection to the past and future. Questions to access connection to past and future include the following:

- Do you feel you are making your ancestors proud?
- Do you use traditional language?
- When was the last time you listened to an elder?

Last, *connection to your better self* signifies our ongoing effort to become a better person for our families, community, and *lāhui*. Any situation in which we find ourselves, even if we are ill, is an opportunity to manifest our better selves. Participants shared stories of friends and family who had cancer or were hospitalized and “in spite of physical ailments, [they were] healthy in spirit, soul, and in many other ways.” The act of reflecting upon and sharing our own stories, happy or traumatic, helps us understand and heal ourselves. Other practices that deepen this connection include finding and sharing our gifts and being aware of what we put into our bodies. Questions to help access connection to your better self include the following:

- What is the story of your name?
- When was the last time you ... cried? Laughed hard? Felt proud?

Woven throughout every story was the importance of spirituality. We considered adding spirituality as another connection but ultimately decided that spirituality should not be separated into a distinct domain because it is embedded in everything—in every connection, relationship, and intention. This fact underscores how the 4 domains are not separate but are interconnected. Each connection is necessary; no 1 alone is sufficient for health and happiness. This interconnectedness underscores the need for spaces and practices where people can make multiple connections. One participant shared the following related story:

When I see people working together on the ‘*āina*, or in a fishpond, or gathering *limu* (seaweed) and doing traditional practices, I actually see them get physically stronger. They get connected to who they are, and to each other. And they feel power—not power over others, but power and control over their own destiny.

**FIGURE 1:**
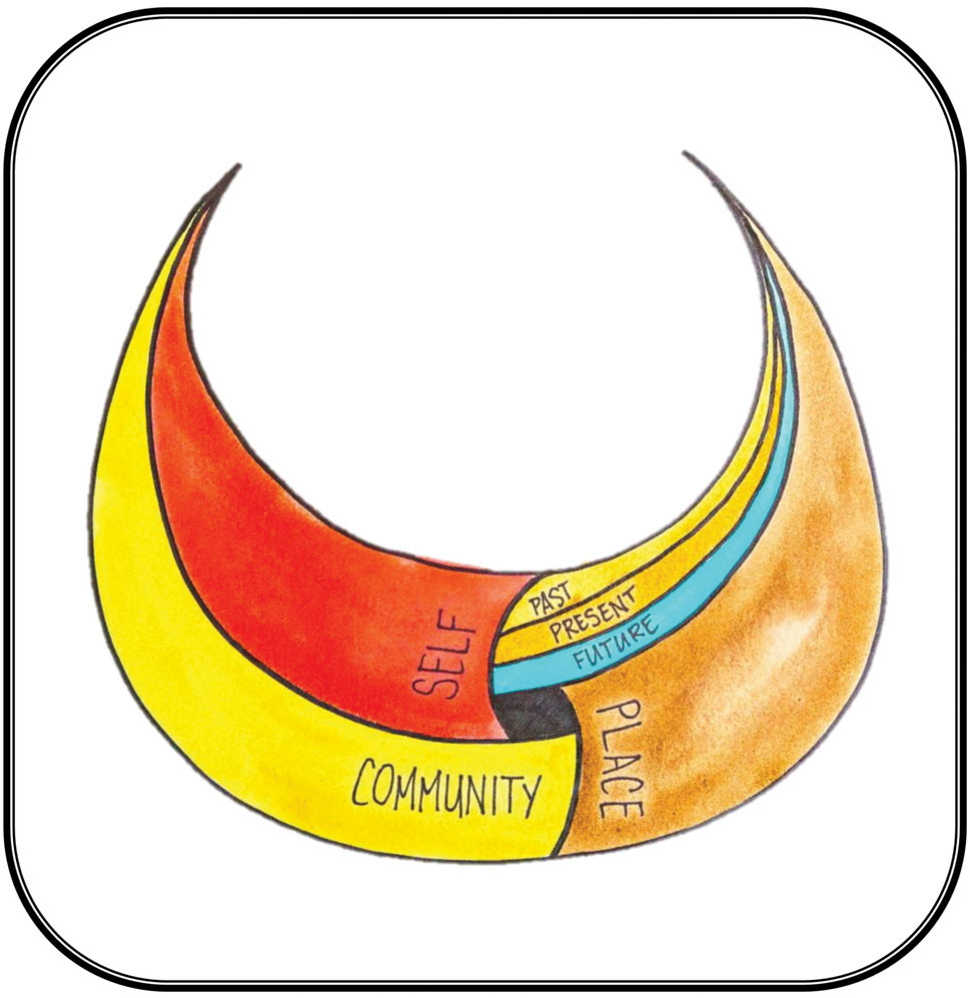
Pilinahā: The Four Connections Framework — An indigenous perspective to health and healing.

Figure 1 illustrates this interconnectedness. Although we describe these connections as distinct but interconnected, they are in fact different aspects of a single wholeness that we aspire to in our lives, families, and communities.

Figure 1 represents the Pilinahā framework. The overall shape is a *hoaka*, a crescent moon representing a full bowl. It is a symbol of abundance. Traditionally, this kind of moon indicates abundance in the land and ocean, and in the framework it refers to the collective abundance of our connections. The 4 woven elements of the framework—community, best self, past–present–future, and place—create a whole, with the open space in the center, intentionally left unnamed, as the interconnecting element of spirituality.

Created by Puni Jackson, the painting in Figure 1 depicts the interconnecting connections to self, community, place, and past and future of the Pilinahā framework.

## Implementation

The foundational stories and practices of Pilinahā are not new. Pilinahā did not transform KKV's community programs. These programs were already led and designed by indigenous people and steeped in the 4 connections. Roots programming is based on the Native Hawaiian belief that food represents our ancestors and our gods; that as we eat, we are ingesting all the good qualities of our ancestors; and that as we work the land to grow our food, we do it with great respect and intention because in so doing we take care of our ancestors, who in turn will care for us. This approach is highly complementary to other Pacific and Pacific Rim peoples in the valley, including Chuukese, Marshallese, Samoans, Filipinos, and Okinawans, who also connect to ancestors and traditional healing practices through Roots food-based programing and its long-standing emphasis on using food to deepen connections to best self, community, past and future, and place.

What was new and innovative for KKV was how we used Pilinahā as a framework to begin evaluating and reflecting upon all our program activities, objectives, and outcomes *more intentionally*. As a formal lens, Pilinahā reminds us that we perpetuate the practices of our grandmothers not out of habit or sentimentality but, rather, because these deep healing practices keep our communities strong. To illustrate these points, we include the following paraphrased evaluations by two Native Hawaiian community members who completed their dietetic rotations at KKV community and clinical programs, including Roots Café and Hoʻoulu ʻĀina:

I learned things at KKV that college didn't teach me. I saw the commitment to listening to the community. I witnessed a holistic approach to patient and community care. I saw how the social determinants of health can be addressed through many venues including integrative medicine, hands on work on the farm, and serving delicious local lunches at the Roots Café. I learned the importance of both the clinical aspects of nutrition as well as the cultural practice of building connections between the health of the land and the health of the people.

I participated in the practice of Aloha circles before important meetings to bring staff together. We chanted, prayed, and shared personal feelings and tears with each other. This is something that's not very common anymore, especially at the workplace. Our society today is numbing its feelings; we don't listen to ourselves anymore, because at times the feelings are painful. Roots Café is helping us reconnect with our ancestral roots. This reconnection taught me it was okay to be myself. That everyone's voice is welcomed, which in turn helped me find my way home.

Using Pilinahā, we see here how the interns made connections with their coworkers and their community, their better selves, the land, and their genealogy.

As we work to understand the ways we already practice the values of Pilinahā, we strive to strengthen this practice and incorporate Pilinahā more widely across the whole organization. Our recent efforts include integrating Pilinahā into program evaluation, personnel assessments, staff development, and staff wellness and healing activities. For example, we have begun conducting an organizational-wide 4-hour curriculum for all of our staff to experience Pilinahā. The director of Ho'oulu Āina leads staff through a series of activities to uncover and share the connections and disconnections in their own lives and the lives of their patients and program participants. It is a way to talk in a safe space about the trauma of disconnection and healing through reconnection. Many KKV staff reported how this curriculum helped them feel “seen” and to “see others” better and to feel “connected,” “loved,” “valued,” and “reinvigorated”—reminding them of the important work they are doing. One participant reflected how “it reminds me about my passion for my work, and why it feeds my soul.”

Participants reflected on how Pilinahā also helped them better reconnect their patients and program participants to healing services. For example, 1 KKV physician shared a story of how the 4 connections shaped his delivery of health care. As a young doctor, he had a Samoan patient with poorly controlled diabetes:

I was concerned for her health prospects and so I passionately told her all the horrible things that could happen to her. She patiently listened to me, nodding her head but not saying much. When I was done, she pulled out photographs of her grandson and explained how proud she was that her grandson was graduating high school and would be the first one in her family to go to college. At the time, I interpreted this abrupt change in topic as the patient's denial of the seriousness of her disease. Now I understand that she was trying to share what was important to her, what she valued, and her source of meaning and health.

When asked what he would do differently if he could go back to that day with his current understanding of Pilinahā, he replied:

I would listen more intently. I would engage her in a more human way. After listening to her values maybe I would explain my values as her doctor, so we could have an honest mutual understanding of each other. In my career, I have been lucky to have had many loving Samoan grandmas patiently teach me that only after I know my patients’ values can I begin to understand their pathways to healing.

As an ongoing practice, many KKV program leaders hold space for participants to reflect on their experiences throughout their programs. Prompts are open ended and include the following: “What made you participate in the program?” “Please share any insights, transformations, or issues you had during the program”; “What does ‘aipono mean to you?” and “What challenges or barriers do you face and what kind of support would help your practice of ‘aipono?” When possible, we have KKV staff scribe participant reflections. Reading through these documents, with the Pilinahā framework in mind, we clearly hear the 4 connections mentioned in participants’ reflections, without specifically prompting them to talk about connections.

For example, 1 woman in our Native Hawaiian birthing group explained that through this program she now understands “feeding my family healthy foods is not only important to my family, but to our *lāhui.*” Other participants also spoke to learning about the importance of food, “being wiser about what we eat . . . choosing how and what we do. How it affects us now, in the future, and possibly how our *kūpuna* (ancestors) previously did . . . it makes us re-think whatever it is we're going to do.” Whereas epigenetic studies of native people most often highlight the genetic perpetuation of negative colonial impacts, these ancestral approaches to food and nutrition follow an ancient example of setting genetic intentionality around love, spirituality, and connectivity in a society.

Participants in our Ehuola program explained “how big” the program was in its “holistic” coverage. Ehuola aims to engage children and their families in food-focused learning, maintain connection to land and their communities, and increase community food productions while highlighting Native Hawaiian cultural practices. The importance of connection was a reoccurring theme in participant reflections. One Ehuola parent shared the following:

I think for me the thing that stands out the most about these past couple years in Ehuola is the community of people that you get to grow with. And the relationships that the kids are building around this particular content area. So that they can have support. As they grow older they know that they're not the only ones trying to live this way, there's a whole community of people who are trying to . . . their friends and with the guidance of their leaders, who they really look up to also.

## Next Steps, Limitations, and Conclusions

When we began this endeavor, we had no thought of publishing our findings. Our sole intention was to better understand our community and our work. After creating Pilinahā from the stories of our first 4 meetings, we continued over 3½ years to pilot test and operationalize the framework. To this day, we continue to integrate Pilinahā into our organizational voice, to use it as a lens to evaluate our programs, and to share it with KKV staff and the many hundreds of students, residents, and volunteers who rotate through our programs, to promote self and communal healing. Outside of KKV, Islander Institute and KKV have used Pilinahā as a framework for convening a variety of organizations throughout the state, including insurance companies, foundations, universities, and nonprofits. The events focused principally on how to shape our health system around what matters most to communities. During this testing phase, we saw how the framework resonated widely. We saw how the 4 connections spontaneously surfaced even during single meetings with indigenous and island people. We saw how during facilitated conversations surrounding people's personal connections and disconnections to place, self, community, and past and future, transformations began to take shape, including participants *1*) forming stronger bonds with one another, *2*) remembering what is important to them, and *3*) being inspired to share these connections with others. Witnessing these reactions motivates us to share Pilinahā more widely.

Yet we recognize its limitations. We have no reason to believe Pilinahā applies to every community. We prefer that people engage their own community processes and clarify their own perspectives and frameworks for health, which may or may not resemble Pilinahā. Also, much work remains to be done to refine Pilinahā, including working out how it addresses spirituality, how it translates into more intentional practice in more varied programmatic and clinical settings, and how it may have unforeseen applications.

Even with these limitations, we are encouraged by Pilinahā thus far. By planting the seed of valuing connections, we have witnessed multiple instances of personal healing in participants as well as stronger intercommunity relationships that continue to develop and bear fruit. This fruit includes other organizations using Pilinahā in myriad ways. It was integrated into health professional curriculum for students of public health and nursing and for state health employees. It is being employed by a national facilitation organization to bridge conversations between federal, state, and native communities around land access and usage. In addition, a local foundation is currently interested in integrating Pilinahā into its grant-making criteria.

We hope that by exchanging more stories with others, we can continue to build the tool kit of questions, practices, and metrics for applying Pilinahā. We are especially interested in metrics that focus on those connections we wish to grow, rather than those that overemphasize the development of more health care services. We envision a health system that upholds the gifts of all providers—clinical professionals and traditional healers alike—and all places, be they hospitals or *mālama āina* programs. (To this end, we are especially inspired that Pilinahā’s development, as a framework of organizational transformation at KKV, was spearheaded by the Director of our Roots program, whose passion and training in both nutritional science and Native Hawaiian culture were instrumental.) We wish for people to see clearly their healing opportunities available through the company of others, through the places and lands they call home, through their histories and traditions, and through their sense of value in whatever context they find themselves. We aspire for health to be an attainable ideal in which people recognize their agency. Pilinahā speaks to the enormity of wisdom and knowledge developed over many generations by cultures and people who lived sustainably for millennia. Our intention is to continue to honor and uplift this insight. From their stories, sparks fly that we hope will re-inspire our systems to hold the dreams and health of all peoples.

## Supplementary Material

nzz001_Supplemental_Appendix

## Notes

The authors reported no funding received for this study.

Author disclosures: SKO, PJ, DD, MKI, and AHA, no conflicts of interest.

Published in a supplement to *Current Developments in Nutrition*. This article appears as part of the supplement “Proceedings of the First and Second Annual Conferences on Native American Nutrition,” sponsored by the Shakopee Mdewakanton Sioux Community's Seeds of Native Health campaign through a gift to the University of Minnesota. The guest editors of the supplement, Treena Delormier and Mindy Kurzer, have no conflicts of interest. The opinions expressed in this publication are those of the authors and are not attributable to the sponsors or the publisher, Editor, or Editorial Board of *Current Developments in Nutrition*.

Supplemental Appendix A is available from the “Supplementary data” link in the online posting of the article and from the same link in the online table of contents at https://academic.oup.com/cdn/.
