# Supplementary material for: Pilinahā: An Indigenous Framework for Health
Source: Curr Dev Nutr. 2019 Feb 22;3(Suppl 2):32–8. doi: 10.1093/cdn/nzz001 (PMC6700459; doi:10.1093/cdn/nzz001)
Supplement: nzz001_Supplemental_Appendix [file nzz001_supplemental_appendix.docx]

**Appendix A: Example of questions for accessing connection and practices for deepening the connection to the four areas of Pilinahā**


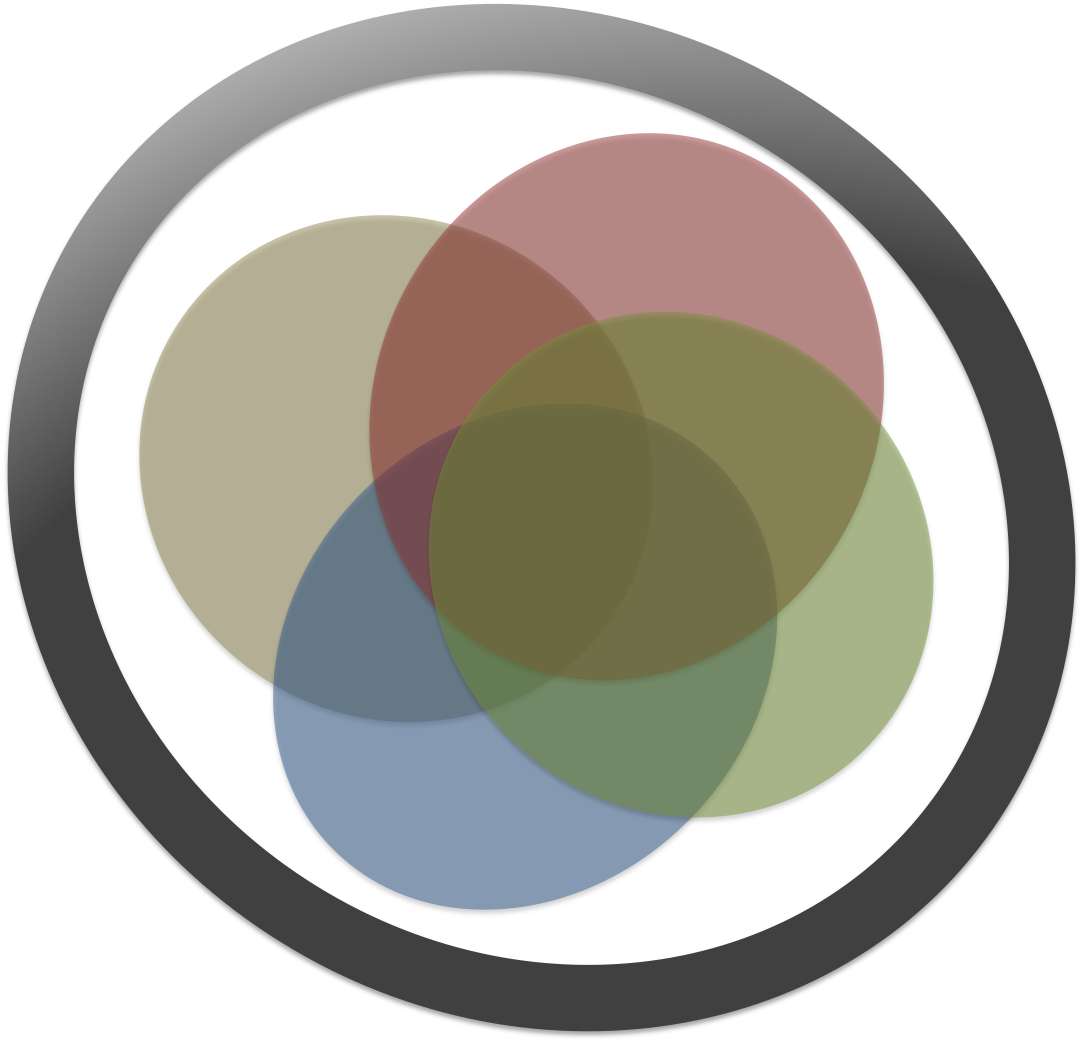
Connection to place

To have a kinship with ‘āina

Examples of Questions for Assessing Connection to Place

- What is the story of the place you call home?
- How well do you know the people and places around you; the “map” of your place?
- What places are special to you?
- Do you have a place where you feel your best—a healing place?
- Can you access the places you need to be in?
- Do you feel a comfort and connection to the built structures around you?
- Do you do any activities in nature? Hands in the earth; being in the ocean, etc.?

Examples of Practices for Deepening the Connection to Place

**Aloha ‘āina – Deepening your love and appreciation for the land**

- *“There is something different about being Native Hawaiian in Las Vegas; not being connected to our ‘āina impacts our sense of self”*
- *“When my father’s been away from home for a while, instead of first spending time with me, he first goes to Ka‘a‘awa and jumps in the water. That’s how he reconnects, that’s how he rejuvenates his spirit.”*
- *“When asked, ‘how are you feeling?’ we often say things like ‘haven’t hit the waves long time’ or ‘haven’t hiked the mountains lately.’”*

**Mālama ‘āina – Caring for and healing the land**

- *“Our ‘ohana incorporate our values and practice in our day-to-day life. We work in our kalo lo‘i every week. Everyone has their own lo‘i to work on. It helps our kids develop critical thinking.”*

**‘Āina, that which feeds us – Food from your land connects you to your land**

- “Our Hawaiian identity is tied to the food we eat, which is connected to place. When I was on Kaho‘olawe, there was lots of fish but we couldn’t really harvest that fish, which was kind of sad.”
- “In indigenous populations that I’ve worked with, you see that when you destroy the population’s food systems, the health problems begin.”

**Mapping – Taking stock of the important points in your surroundings**

- *"In preparing for the next disaster, people knew the locations of every person who would need extra help; where they lived and what their needs were.”*


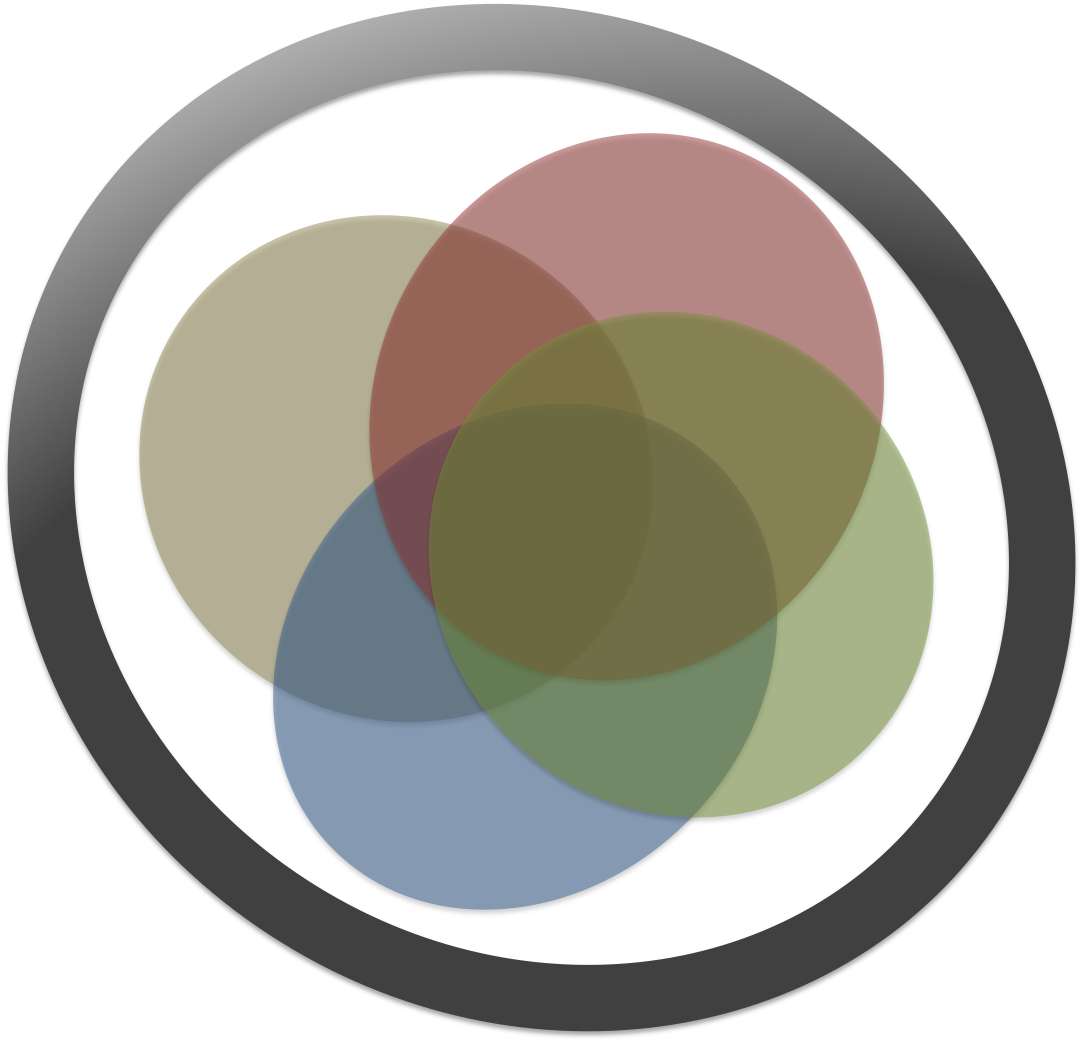
 Connection to community

To love and be loved; To understand and be understood

Examples of Questions for Assessing Connection to Community

- What is the story of a person you feel closest to?
- Do you get good quality time with your ‘ohana?
- Do you have someone you can trust and turn to when you need help?
- Is there someone who trusts you and turns to you when she/he needs help?
- Do you feel accepted by others and are you accepting of others?
- Do you feel good about the people you work with?
- Who are the people you eat with?
- Have you told someone you love how much they mean to you?

Examples of Practices for Deepening the Connection to Community

**‘Ohana style – Good health flows through relationships**

- *“We did a diabetes project in Hana. It was ‘ohana style – the mo‘opuna, the dog – after awhile, everyone wanted to come. We would sit down with them and talk story. The husbands came to realize that it wasn’t just about their wives illness, but that they had to help their wives, be supportive of them. “…“Some of the couples said they came to the sessions because it was like date night for them.”*
- *“… what we are talking about, is relationships. If I cannot talk to my kids, why am I trying to be healthy? No make sense. I tell my kids, no put me in a home.”*

**Generosity, giving**

- *“My parents don’t necessarily have expectations of a longer life. They are happy, generous, doing good for others. We still need pathways to health but we shouldn’t place judgments on how others live their values.”*
- *“Uncle Eddie asked – would you die for your patient? That’s the level of sacrifice.”*

**Starting where people are at**

- *“There was a patient that I had and he was going blind. I kept trying to talk to him about what his health situation was about but he only wanted to talk about his grandson graduating. I started to listen to his story about his grandson and eventually we got to talk about his own health.”*

**Skin-to-skin**

- *“My sister has a one-month old baby. In order for her to produce breast milk, she needs to have skin-to-skin contact with her baby. That’s what we’re trying to do with our work. When we’re skin-to-skin with each other, we create memories, we remember, we become hungry, and we feed each other’s hunger.”*
- *“In this circle of friends, I feel an absolute sense of safety… like that feeling I had as a child curled up in my grandfather’s arms.”*

**Talk story**

- *“When we were working together on the pili—talking story—time was abundant.”*
- *“We were crying one minute and laughing the next. Each of us shared an event in our lives that had a transformational moment… and we’re okay with ourselves.”*


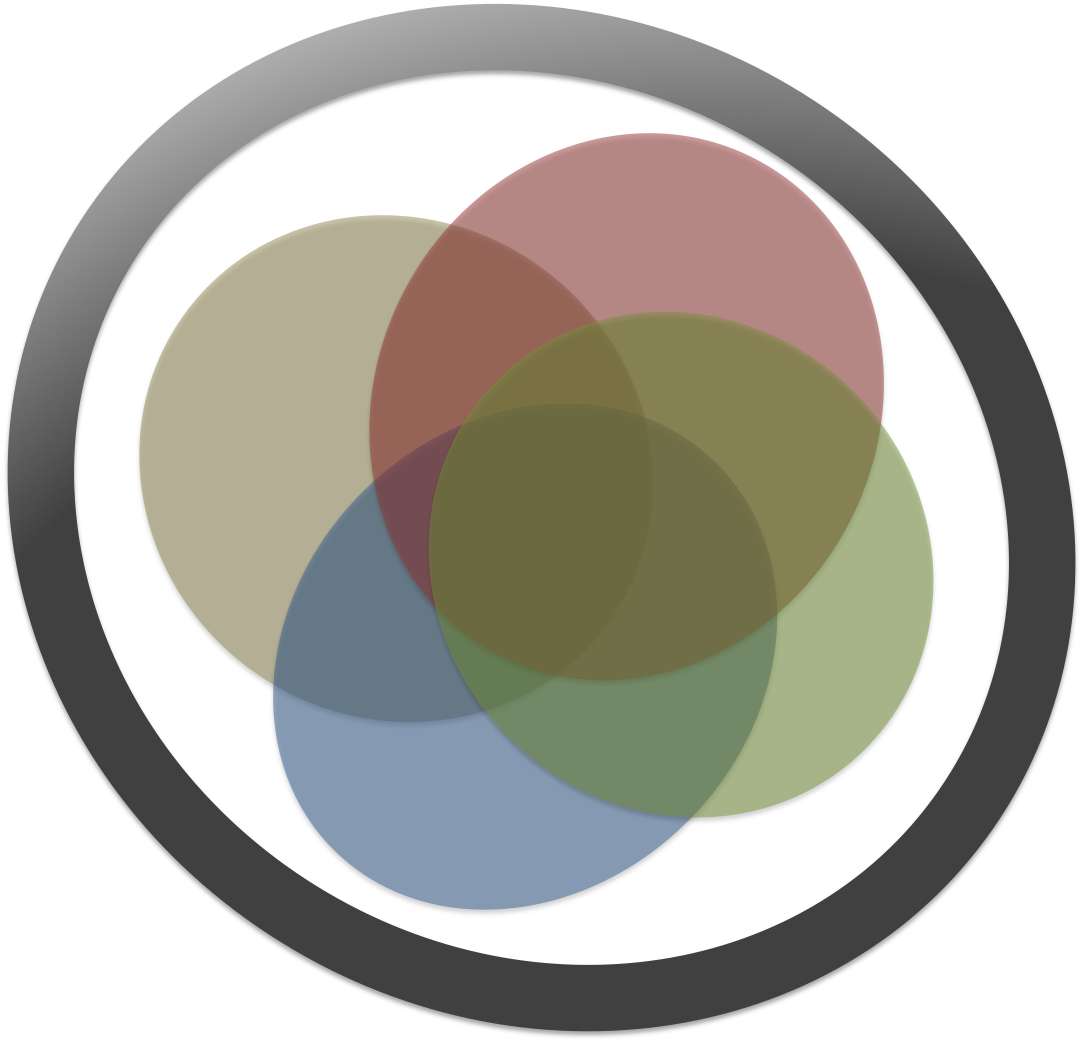
Connection to past and future

To have kuleana; a purpose in the world

Examples of Questions for Assessing Connection to Past and Future

- What is the story of your ancestry?
- Do you feel you are making your ancestors proud?
- Do you use traditional language?
- If you think about the past and future as chapters in a book, what is your part in that story?
- When was the last time you listened to an elder?
- What do you do for the future generations?
- When was the last time you held a baby; or read to a child; or listened to a teenager?
- What is your happiest memory of someone who has passed on?

Examples of Practices for Deepening the Connection to Past and Future

**Cultural understanding and appreciation**

- *“The fact of our genealogies makes us whole. It’s an unbroken chain of stories and practices.”*
- *“Our ancestors were a healthy people.”*
- *“On Hokule‘a’s World Wide Voyage, we came across a people that didn’t have much, but they have everything! Some people are embracing the pearl industry to create economic opportunities. The kūpuna there, however, don’t see pearls as the way to wealth. They understand the value of carrying on wa‘a traditions; that they have clean water and land; that they need to feed not just their body but their spirit also. They determine for themselves what is health, what is wealth – not let other people’s standards, frames, define that for them.”*

**Carrying on traditional practices – Experiencing the wisdom of our ancestors**

- *“When we’re on a long voyage – there’s routine on the wa‘a. We have a groove – we can only study, sing, talk story. When we come off the canoe, we get sick. Too many choices, questions.”*
- *“At one of our community workdays, the Chuukese uncles saw adzes and went to town helping to carve the canoe. The uncles, through work on the canoe, were able to feel comfortable in their bodies, to remember their connection to their parents and grandparents. Where do these uncles find this chance to connect where they live in public housing, in their day-to-day life?”*


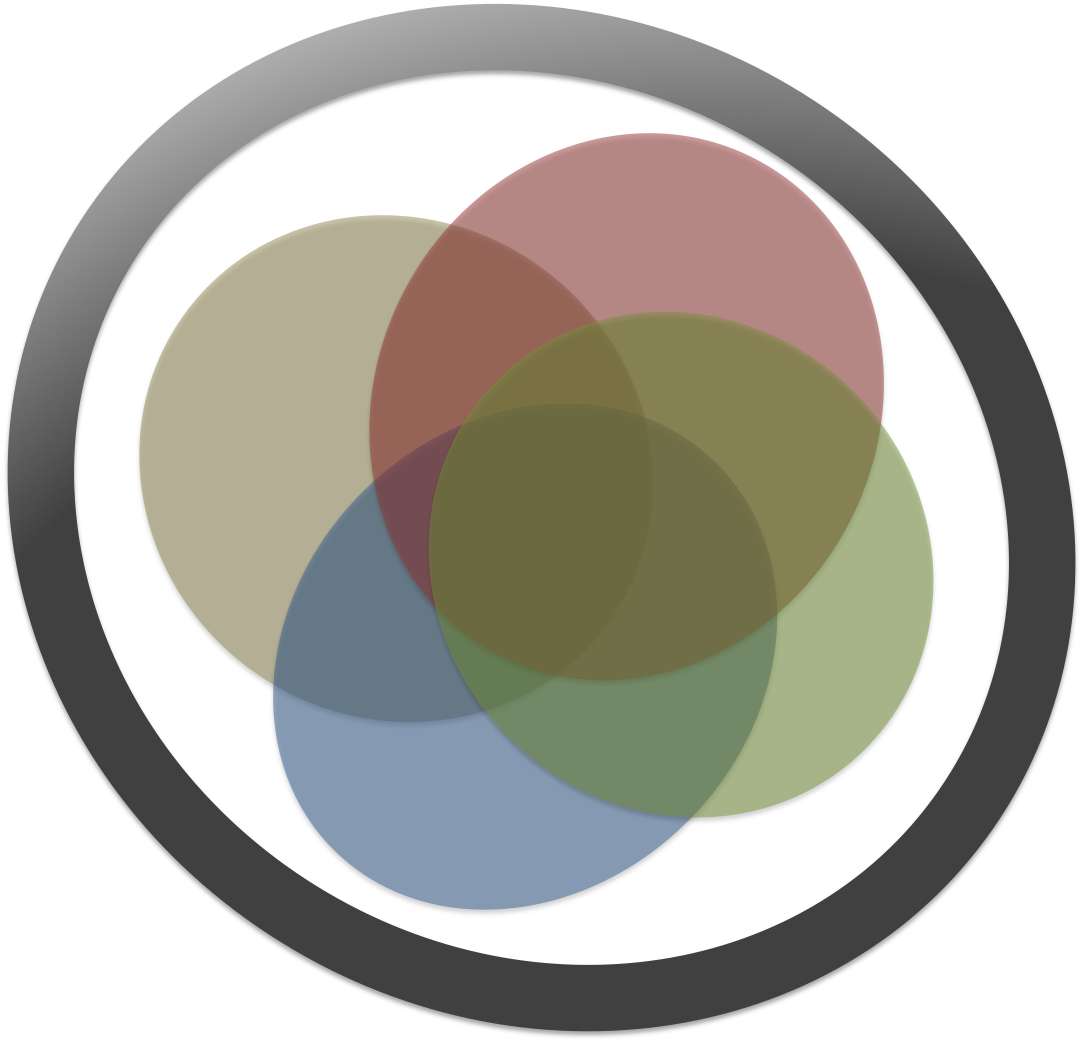
 Connection to your better self

To find and know yourself

Examples of Questions for Assessing Connection to Your Better Self

- What is the story of the last time you felt healthy?
- When was the last time you felt really good about something you did?
- Do you feel good about the food you are putting into your body
- Do you understand how medicines you take and medical procedures you’ve had work?
- Do you feel good about the roles you play? At home? At work?
- Are you getting enough sleep?
- When was the last time you… Cried? Laughed hard? Felt proud? Learned a lesson?
- What is the story of your name?

Examples of Practices for Deepening the Connection to Your Better Self

**Finding your gifts, living by your values**

- *“At Ho‘oulu ‘Āina, staff are encouraged to discover their gifts first. Often they react angrily, become confused and anxious. Can become frustrating. But as they persist, the frustration becomes a source of learning. The ‘kalo walk’ was the result of frustration leading to learning, experimentation, and innovation.”*

**Wholeness/health in the midst of “illness”**

- *“(I met a doctor) who was exceptionally generous in the midst of the limitations of him being quadriplegic. Even when he was hospitalized, he invited visitors in and spent time to lift their spirits up and provide guidance to young people. In spite of his physical ailments, he was healthy of spirit, soul, and in many other ways.”*
- *“…she had cancer – and yet the way she was able to deal with it and feel happy and ‘healthy’ throughout was incredible.”*

**Awareness of what we put into our bodies**

- *“I normally eat healthy but my family eats macaroni salad, red hot dogs – so I would get sick when I go over to eat. I used my getting sick as an opportunity to slow-by-slow help them change their eating habits. Taught my aunty how to use and eat olive oil!”*
- *“My daughter needed to bring a salad to school but I didn’t have the time to help her make it. So I told her that I’d pick up a salad at Whole Foods the next morning. After being frustrated with me about this, she told me, ‘But mom, there’s no mana in the salad at Whole Foods!’ We made a small salad together that night.”*

**Story itself is health – If you delve deep into story, you will find your truth**

- *“My friend’s brother was murdered and she didn’t share about it until the end of a poetry event that we had. It really helped her to be able to open up about it. People need spaces so they can tell their story. Our inability to share story can lead to us becoming sick.”*
- *“*How can we create spaces for sharing our story, raising questions for ourselves and each other that help to put us on pathways to an awareness of our kuleana.
